# Genomic regions with distinct genomic distance conservation in vertebrate genomes

**DOI:** 10.1186/1471-2164-10-133

**Published:** 2009-03-27

**Authors:** Hong Sun, Geir Skogerbø, Xiaohui Zheng, Wei Liu, Yixue Li

**Affiliations:** 1 Key Laboratory of Systems Biology, Shanghai Institutes for Biological Sciences, Chinese Academy of Sciences, Shanghai, PR China; 2 Biological Technologies, Wyeth Research, Cambridge, Massachusetts, USA; 3 Shanghai Center for Bioinformation Technology, Shanghai, PR China; 4 Bioinformatics Laboratory and National Laboratory of Biomacromolecules, Institute of Biophysics, Chinese Academy of Sciences, Beijing, PR China; 5 Zhongxin Biotechnology Shanghai Co Ltd, Shanghai, PR China; 6 Hetian Peoples' Hospital, Xinjiang, PR China

## Abstract

**Background:**

A number of vertebrate highly conserved elements (HCEs) have been detected and their genomic interval distances have been reported to be more conserved than protein coding genes among mammalian genomes. A characteristic of the human – non-mammalian comparisons is a bimodal distribution of relative distance difference of conserved consecutive HCE pairs; and it is difficult to attribute such profile to a random assortment. We therefore undertook an analysis of the human genomic regions confined by consecutive HCE pairs common to eight genomes (human, mouse, rat, chicken, frog, zebrafish, tetradon and fugu).

**Results:**

Among HCE pairs, we found that some consistently preserve highly conserved interval distance among genomes while others have relatively low distance conservation. Using a partition method, we detected two groups of inter-HCE regions (IHRs) with distinct distance conservation pattern in vertebrate genomes: IHR1s that are bordered by HCE pairs with relative small distance variation, and IHR2s with larger distance difference values. Compared to random background, annotated repeat sequences are significantly less frequent in IHR1s than IHR2s, which reflects a correlation between repeat sequences and the length expansion of IHRs. Both groups of IHRs are unexpectedly enriched in human indel (i.e. insertion and deletion) polymorphism-variations than random background. The correlation between the percentage of conserved sequence and human IHR length was stronger for IHR1 than IHR2. Both groups of IHRs are significantly enriched for CpG islands.

**Conclusion:**

The data suggest that subsets of HCE pairs may undergo different evolutionary paths in light of their genomic distance conservation, and that sets of genomic regions pertain to HCEs, as well as the region in which HCEs reside, should be treated as integrated domains.

## Background

Comparative sequence analysis has become an essential component for studying genome function. Genome-wide comparison of human and rodents DNA sequences discovered more than 5,000 ultraconserved elements (UCEs) of absolute identity and >100 bp in length [1], of which 77 percent were located outside annotated exons, and long-range comparison between man and pufferfish also identified high numbers of similar genomic elements [2,3]. A common characteristic of these highly conserved elements is their strong tendency to occur in clusters along the mammalian chromosomes [1-4] with their relative order as conserved as that of protein coding genes [5]. A relative distance difference (RDD) measure was defined to evaluate the genomic distance change ratio of pairs of UCEs found at adjacent positions in the human, mouse and dog genomes, showing that the genomic distance between such elements is significantly more conserved than corresponding genomic distance between orthologous protein coding genes [5]. An intriguing observation is that the conservation of the genomic distance between pairs of UCEs [1] also exists in the preliminary analyses of evolutionarily more distant vertebrates, and displayed a distinct pattern with a fraction of UCE pairs displaying nearly unaltered genomic distances over long evolutionary distances and a second fraction of UCE pairs with less conserved genomic distances [5]. It is hard to imagine that such genomic distance profile could arise by random as it was consistently found in comparisons between several genomes, and suggests that the genomic distance conservation may take on different characteristics corresponding to different genomic regions.

Genomic sequences have been divided into functional blocks based on various characteristics, e.g. biological roles or sequence conservation. Early research works mainly focused on synteny blocks defined according to conserved DNA sequences or orthologous genes to study the evolutionary history of rearrangements in entire genomes [6-8]. Concordant higher-order patterns from functional genomic studies suggest that the human genome and other large genomes are organized into higher-order functional domains [9]. For example, genomic regulatory blocks (GRBs) spanned by highly conserved noncoding elements, developmental regulatory target gene(s), and phylogenetically and functionally unrelated "bystander" genes, are conserved between mammal and fishes [10]. The identification and study of these higher-order functional domains within the human genome is of great importance. Our early observation of distinct two-peak distribution profiles in genomic distance conservation suggested that some other aspect of the DNA sequences adjacent to the UCE pairs may likewise be different, and might thus be treated as two distinct genomic regions or blocks [5].

Different sets of highly conserved elements (HCEs) vary somewhat with respect to their locations in the human genome, some are exclusively found in non-protein-coding sequence (e.g. CNEs [2] and UCRs [3]) and others contain exons of protein-coding genes (UCEs [1]). Though it has been suggested that exonic UCEs represent a distinct subset [11], no satisfactory explanations for the extreme degree of sequence conservation of exonic UCEs have been presented. In our previous study [5], we have shown that with respect to distance conservation there were no substantial differences between different data sets (i.e. UCEs [1], CNEs [2] and UCRs [3]) or between UCE pairs with exonic and non-exonic locations. We have therefore integrated the three datasets [1-3] and undertaken an analysis of the human genomic regions confined by HCE pairs common to eight genomes (human, mouse, rat, chicken, frog, zebrafish, tetradon and fugu).

## Results

In our previous work, we calculated a relative distance difference (RDD [5]; Methods) value to assess whether the genomic distances between consecutive UCEs show less change than that between other adjacent genomic elements (i.e. genes and exons) in the human, mouse and dog genomes. The analysis showed that in addition to an extreme level of sequence conservation, UCEs also display strong conservation of mutual genomic distances among mammalian species [5]. The conservation of distance between pairs of UCEs [1] is also found between evolutionarily more distant vertebrates, but the distributions of RDD values show a persistent nature of distinct two-peak profiles in all mammal – non-mammal comparisons, with one peak close to zero, and another at a more negative value [5]. Low number of UCEs [1] was used in the previous analysis therefore a large data set is warranted to validate these findings.

To facilitate this investigation, we constructed a dataset integrated from three independent works [1-3]. A direct element by element comparison shows that two-thirds of the non-exonic UCEs from data set [1] do not overlap with HCEs from any of the two other data sets [see Additional file 1]. The smallest data set of ~1,400 conserved non-coding elements (CNEs) [2] had the highest fraction of overlaps (~80%) to data set [1] and [3], whereas the set of ultraconserved regions (UCRs) [3] has ~50% overlaps with others. We combined these three published data sets [1-3] to form an integrated data set consisting of 7,570 distinct highly conserved elements (HCEs) in the human genome. We used BLASTn with non-stringent parameters and criteria for order and genomic distance conservation to locate all occurrences of the same HCEs in the mouse, rat, chicken, frog, zebrafish, fugu and tetraodon genomes [see details in Methods; Additional file 2]. The resulted number of orthologous HCEs that can be located uniquely in the different genomes is variable: more than 95 percent of human HCEs could be anchored to the rodent genomes, 71 percent to the chicken genome, and around 24 to 30 percent in fish [see Additional file 3]. From the comparisons with the human genome more than 99 percent of HCEs were found linked with at least one other HCE/HCEs in all other genomes, including the linkage relationship with quite a number of HCEs in the fish genomes. More than sixty percent of HCEs were found ordered together with at least 5 individual elements [see Additional file 4], which indicates the tendency for HCEs to preserve order conservation among vertebrate species.

We calculated RDD values between pairs of HCEs and compared them with RDD values for pairs of genes and exons of these genes. Similar to what has been reported for mammalian comparisons [5], the absolute relative distance difference (|RDD|) were significantly lower for HCE pairs than for pairs of genes or exons [see Additional file 5; Wilcoxons unpaired test, p value < 2.2e-16]. Calculated as absolute values (|RDD|), the median distance difference for HCE pairs in the human-chicken comparisons was 0.46, which is about half that for gene pairs (0.91) and exons (0.95) [see Additional file 5]. The difference between distance conservation of HCE pairs and gene pairs is most pronounced for the human – zebrafish comparison; median |RDD|_HCE _being only 32 percent of median |RDD|_gene_. HCE-HCE absolute distance differences are also significantly less than exon-exon distance differences (within gene); the latter being only slightly different from the gene-gene relative distance differences.

The RDD distribution profiles were also markedly different for the three different pair comparisons (HCE-HCE, gene-gene, and exon-exon). The RDD distributions for HCE pairs show distinct two-peak profiles, with one peak close to zero and another at a more negative value. RDD values for gene pairs, in contrast, show only one peak skewed toward more negative values. The distributions of exon RDD values are wider than for both HCE and gene pairs. The distributions of all three data show a peak at relatively low RDD values (-1 to -2) for all four human – chicken/fish comparisons [see Additional file 6]. However, the distribution of HCE RDD values consistently show an additional, dominant peak around zero, indicating the existence of a subset of HCE-HCE pairs whose distances have been conserved across vertebrate evolution. Even for Fugu and Tetraodon, whose genome sizes are only around 13 percent of the human genome, the result indicates that around 30 percent of the analyzed HCE pairs have largely unaltered distances (i.e. |RDD| within ± 0.116~0.409) compared to the human genome [see Additional file 7].

**Figure 1:**
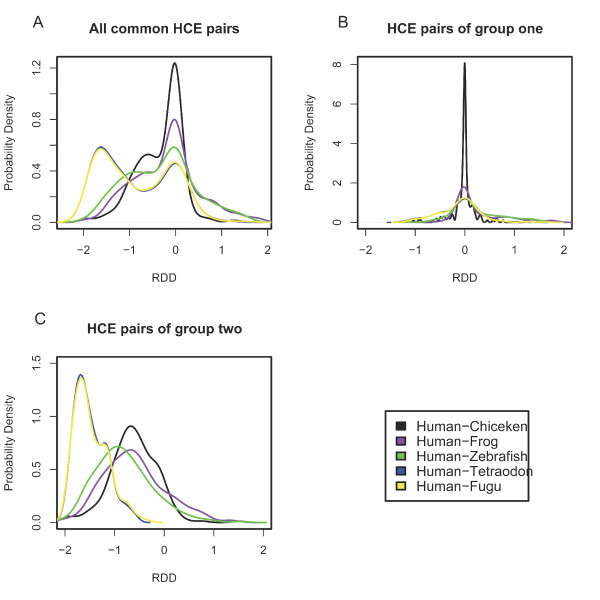
**RDD distribution for HCE pairs common to the five genomes**.

A total of 403 HCE pairs are shared by the five non-mammalian species and human genome. The two-peak distribution profiles of RDD values still persist, with one peak close to zero and another peak (or 'shoulder') at a more negative value, as shown by mapping this integrated data set of HCEs onto the five non-mammalian genomes (Figure 1A). Most of these common HCE pairs are unique and linked with each other in the query genomes as they do in the human genome [see Additional file 8]. HCEs have been reported to be unique and clustered in the human genome [1-3], here we see a similar tendency in the non-mammalian genomes.

### Inter-HCE regions with distinctive distance conservation patterns

**Figure 2:**
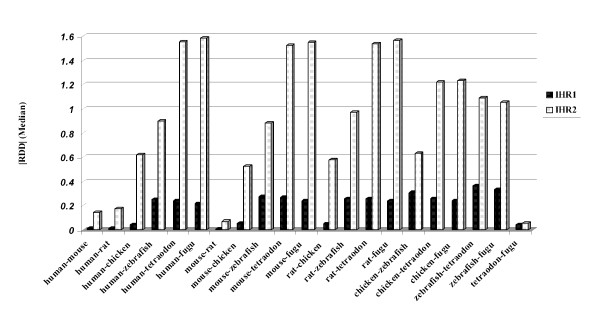
**Median |RDD| for HCE pairs of IHRs**. Median | RDD| of IHR2s were much higher than that of IHR1s for the comparison of any two pair-wise genomes.

In addition to the persistent nature of the two-peak distribution profiles, a remaining question is whether there exist any other characteristics pertaining to the regions confined by the HCE pairs common to the human and five non-mammalian genomes. To test this, we divided the 403 HCE pairs into two groups by using a partitioning clustering method based on the matrix of absolute RDD (|RDD|) values for the human – non-mammalian comparisons (Methods). RDD values of group one HCE pairs are centered around zero (Figure 1B), whereas those of group two are more widely scattered around a more negative value (Figure 1C). The distances between group two pairs (mean 46 Kb) are significantly longer than the distances between group one pairs (mean 2.8 Kb) [see Additional file 9; Wilcoxon test p value = 2.2e^-16^]. The |RDD| value of two consecutive HCEs has been reported to be positively correlated with the distance between the pair [5], we see here a reflection of the same correlation. We call the inter-HCE regions IHRs and subsequently classify the IHRs into two types based on the (above mentioned) partitioning result [see Additional file 10]. We obtained 188 IHRs (termed as IHR1s which are bordered by HCE pairs with relative small |RDD| values), and 215 IHRs (termed as IHR2s which are bordered by two consecutive HCEs with larger |RDD| values). All these 403 HCE pairs are also detected in the rodents. An intriguing observation is that for any pair-wise comparisons among the eight genomes, the median |RDD| values for HCE pairs of IHR2s are constantly much higher than those values of IHR1s [Figure 2, see Additional file 11]. Given the persistent nature of distinct distance conservation of the two groups of IHRs, it is difficult to assume that such profile was the result of a random assortment. Rather, it seems more likely that subsets of HCE pairs may undergo different evolutionary paths in the sense of genomic distance conservation.

We subsequently defined the subset of intergenic IHRs when their flanking HCEs are intergenic in the human genome with no genes in between. For both IHR1 and IHR2 groups, there are more intergenic IHRs than other categories, with 40 percent intergenic IHR1s and 49 percent intergenic IHR2s, respectively (Table 1). We further calculated genomic distances between intergenic IHRs and their closest neighboring genes. Using the distance to the closest gene for statistical analysis, the average distance is 113 Kb for intergenic IHR1s and 150 Kb for intergenic IHR2s (Table 2). A high percentage of intergenic IHRs are more than 10 Kb away from the nearest genes [see Additional file 12].

**Table 1:** Number of HCE pairs with different genomic locations

	Group 1	Group 2	total
Exonic-Exonic	29	16	45
Intronic-Intronic	62	57	119
Intergenic-Intergenic	77	111	188
Exonic-Intronic	16	12	28
Exonic-Intergenic	4	4	8
Intronic-Intergenic	0	15	15

Total	188	215	403

**Table 2:** Distance between intergenic IHRs and their nearest genes.

		Distance (Kb)			
		
	Number	Min	Median	Mean	Max
Intergenic IHR1	75	0.6	85.3	113.2	482.0
Intergenic IHR1 with CpG islands	11	2.5	22.8	60.5	398.1
Intergenic IHR2	106	0.4	113.0	150.8	652.1
Intergenic IHR2 with CpG islands	15	2.9	55.8	92.6	421.2

We also identified a few human genomic regions that are spanned by the same type of IHRs, indicating that the distance variation of HCEs in these regions is probably associated [see Additional file 13]. An intriguing observation is that ten IHR1s are clustered in a region close to 1 Mb, and the corresponding eight HCE pairs are all located in intergenic regions.

### Enrichment of DNA repeat sequences

Human genome has a much greater portion of repeat sequences and it is believed there is a correlation between genome size and repeat content. We therefore asked whether there are any differences in the enrichment of human DNA repeat sequences between IHRs and random genomic regions. As both the number and length of the two groups of IHRs are different, we used randomly selected regions to test the significance. Repeat sequences appear more frequently in IHR2s than in IHR1s. Compared with the sets of corresponding random regions, repeat sequences are significantly less frequent in IHR1s (43 percent, Table 3; p value < 0.001, 74 percent for the random background), but more in IHR2s (97 percent, Table 3; p value = 0.052, 94 percent for the random background). Here, we found a correlation between repeat sequences and the length expansion of IHRs. Fewer IHR1s containing repeat sequences may reflect evolutionary pressure against either transposon-derived sequence in these regions or the distance-distorting effects of inclusion of longer repeat sequences between the bordering HCEs to maintain the shorter IHR1 length.

**Table 3:** Percentage of repeated base pairs within IHRs.

		IHR1		IHR2	
	
		Observed	Expected	Observed	Expected
Percentage of IHRs containing repeat (%)		43 (0.001)	74	97 (0.948)	94

Average percentage of repeated base pairs (%)	SINE	4.26 (0.001)	16.76	11.00 (0.001)	13.51
	LINE	2.37 (0.001)	26.63	13.42 (0.001)	20.96
	LTR	0.60 (0.001)	10.81	4.67 (0.001)	8.53
	Low_complexity	4.86 (1)	0.73	0.72 (0.984)	0.58
	scRNA	0 (0.001)	0.005	0.006 (0.783)	0.004
	DNA	0.75 (0.001)	3.88	2.78 (0.146)	3.06
	RNA	0.03 (0.967)	0.005	0.001 (0.404)	0.005
	srpRNA	0 (0.001)	0.008	0.0008 (0.110)	0.008
	snRNA	0 (0.001)	0.02	0.01 (0.716)	0.01
	tRNA	0 (0.001)	0.003	0.0009(0.219)	0.003
	rRNA	0 (0.001)	0.007	0.005(0.635)	0.006
	Simple_repeat	1.04 (0.476)	1.17	1.02 (0.828)	0.92
	Satellite	0 (0.001)	0.49	0 (0.001)	0.36

Randomly selected human genomic regions were used to test the significance, the fraction of times in which the random sample set scored lower average scores than those of the IHRs provided the basis for the statistical significance. p value was given in the bracket.

We also found that both types of IHRs contain significantly less sequences of SINE (4.3% for IHR1s, 11.0% for IHR2s), LINE (2.4% for IHR1s, 13.4% for IHR2s) and LTR (0.6% for IHR1s, 4.7% for IHR2s) compared to the random backgrounds (Table 3; p value < 0.001); however, both types of IHRs are significantly enriched in low complexity DNA sequences (4.9% for IHR1s, 0.7% for IHR2s) (Table 3; p value < 0.001 for IHR1; p value = 0.016 for IHR2;). We also tested the enrichment of long transposon-free regions (TFRs) in IHR1s and IHR2s. TFRs have been reported to be associated with both protein coding genes and UCEs [12]. Of the 188 IHR1s, 60 percent are intersected with TFRs (2.6% for the random background); and 52 percent of the 215 IHR2s are intersected with TFRs [see Additional file 14; 12% for the random background]. Both groups of IHRs show a significant enrichment of TFRs compared with random selected regions, indicating a complex relationship between TFRs and distance conservation.

### Unexpected enrichment of indel variation

Since HCEs are highly conserved at not only sequence level but also their genomic organization (e.g. order and distance), we suspected that IHRs might not tolerate any large extent of rearrangements. We therefore asked whether there are any differences in the distribution of human indel (i.e. insertion and deletion) polymorphisms in the IHRs.

Mills *et al. *[13] recently identified a set of small indels from three different human populations. As a negative control, we used randomly selected genomic regions with the same number and length of corresponding IHR1s and IHR2s, respectively. The frequency of which the random samples had higher average scores than those of the IHRs provided the basis for the statistical significance. None of the IHR types are deleted in small indels, and IHR2s are actually significantly enriched. We found that 16 percent of IHR1s (30; p value = 0.241, 27 for the random background) versus 81 percent of IHR2s (174; p value < 0.001, 156 for the random background) contain small indels [Table 4; see Additional file 15]. Both results are not in accordance with the expected. Considering the highly conserved length of IHR1s, less IHR1s are expected to contain indels than random background; and as many IHR2s as random selected regions are expected to contain indels. For the regions with indels, we calculated the percentage of insertion/deletion base pairs over the whole length of corresponding IHRs, and found no significant differences in both types of IHRs compared with the randomly selected human genomic regions [Table 4; see Additional file 15]. Previous works have suggested that the genome-wide indel rates are not uniform and that indel events are not neutral [14]. Investigations of human indels indicated that most indels have arisen from the most recent variation events [15,16]. In spite of the observation of overrepresentation of indels in human IHRs, the fact that the length of IHRs remains highly conserved among vertebrate genomes than the distance of gene or exon pairs suggests that the distance between consecutive HCEs is under high selection pressure and is important for HCEs to exert their biological function.

**Table 4:** Enrichment of human indels within IHRs.

	IHR1		IHR2	
	
	Observed	Expected	Observed	Expected
Number	30 (0.241)	27	174 (0.001)	156

Average percentage of deleted base pairs (%)	4.53 (0.107)	1.66	0.49 (0.226)	0.39

Average percentage of inserted base pairs (%)	0.03 (0.679)	0.05	0.01 (0.801)	0.01

Randomly selected human genomic regions provided the background to test the significance, p value was given in the bracket.

### Conserved sequences within IHRs

A previous observation is that |RDD| and sequence conservation are to some extent positively correlated [5]. We used the datasets of phastCons elements provided by the UCSC online server to test the conservation characteristic within the IHRs. As for Tetraodon and Fugu, there are presently no phastCons data from the UCSC online service, so these two genomes were excluded from the sequence conservation analysis.

**Figure 3:**
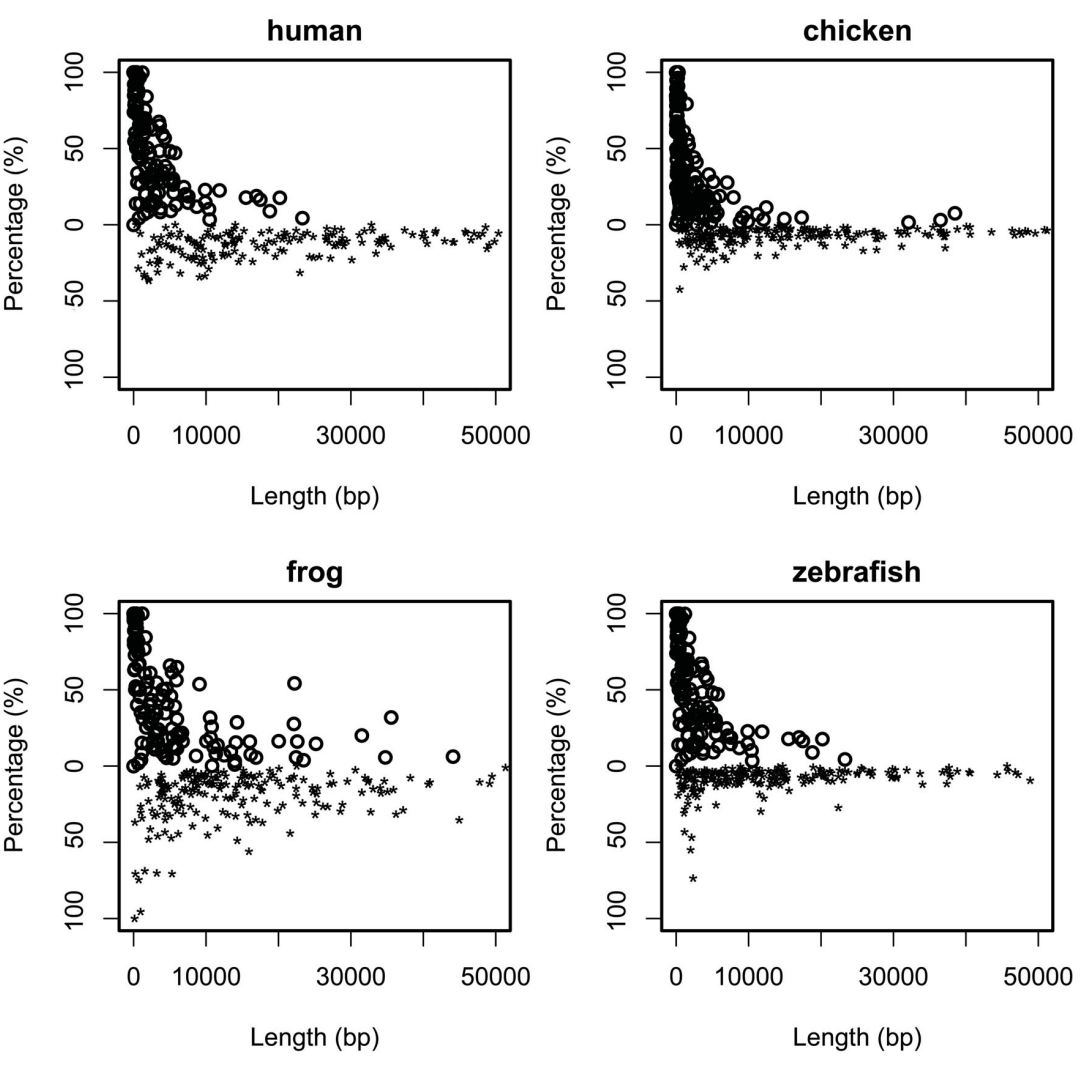
**Correlation between the percentage of conserved sequence and the length of the two groups of IHRs**. Circles represent the data for IHR1 and stars for IHR2.

The correlation between the percentage of conserved sequence and human IHR length is stronger for IHR1 than for IHR2. The conservation percentage is below 50 percent in almost all IHR2s, even in short IHR2s with length close to IHR1s (Figure 3). Among the IHR1s, some have a high percentage of conserved DNA sequence, whereas others not. Considering the generally high degree of distance conservation of the IHR1s, their length might have been under a higher level of evolutionary constraint than the DNA sequences within the regions.

In the human genome, the average length of conserved elements is nearly the same in IHR1s and IHR2s (73 bp for IHR1s and 76 bp for IHR2s, respectively; Table 5). However, the average inter-distance between two consecutive conserved elements of IHR2s is almost 2.8 times longer than the IHR1s (327 bp for IHR1s and 894 bp for IHR2s; Table 5), thus resulting a lower average sequence conservation for IHR2s. The same tendency was found for the other three genomes. The phastCons element data were derived by a multiple species alignment algorithm [17], and the length of the same conserved fraction vary little across the compared species and therefore contribute little to the distance differences between species. No significant differences was observed in the length distribution of conserved fractions between the two groups of IHRs in the human genome (Table 5; p value = 0.1798), indicating that the same length of potential functional sequences with lower sequence conservation occupying the space of both groups of IHRs.

**Table 5:** Length of conserved fractions and distance between two consecutive conserved fractions within IHRs.

			Length of conserved fractions (bp)		Distance in between two consecutive conserved fractions (bp)	
			
		Total number of conserved fractions	Median	Mean	Median	Mean
Human	IHR1	1460	37	73	54	327
	IHR2	10041	39	76	100	894
	p value (IHR1, IHR2)		0.1798		2.2e-16	

Chicken	IHR1	727	37	65	50	569
	IHR2	3504	42	72	74	1302
	p value (IHR1, IHR2)		0.0020		8.3e-11	

Frog	IHR1	576	158	233	140	867
	IHR2	2531	156	226	249	1463
	p value (IHR1, IHR2)		0.6452		6.2e-06	

Zebrafish	IHR1	560	65	96	144	903
	IHR2	1863	73	104	241	1686
	p value (IHR1, IHR2)		0.0018		2.3e-05	

Two samples Wilcoxon test was used to test the significance.

### IHRs and CpG islands

Both groups of IHRs are significantly enriched for CpG islands compared with the corresponding random backgrounds in the human genome: about 10 percent of IHR1s (0.5% for the random background) and 14 percent of IHR2s (2.3% for the random background) were found to contain CpG islands [see Additional file 16, Additional file 17; p value < 0.001]. We further tested the percentage length of CpG islands and observed the difference: average 45% for IHR1s and 7% for IHR2s. The percentage length of CpG islands between IHR1s and IHR2s is significantly different [see Additional file 16; Wilcoxon test, p value < 5.5e-06].

For both IHR1s and IHR2s with CpG islands, the pair-wise genomic loci of HCEs are only significantly sparse in the "intronic-intronic" class [see Additional file 18; Hypergeometric test, p value = 0.0024], which can easily be understood that there are exonic sequences residing in between the HCEs and that promoter elements (i.e. CpG islands) are less likely to be located in the exonic regions. We next checked the environment of those intergenic-intergenic IHRs with CpG islands, eleven/fifteen intergenic IHR1s/IHR2s were found with CpG islands, respectively (Table 2). Eight intergenic IHR1s with CpG islands are more than 8 Kb away from the closest gene. Fifteen IHR2s with CpG islands are located in the intergenic regions; only three reside in the regions less than 10 Kb away from the nearest gene. A high percentage of intergenic IHRs are more than 10 Kb away from the nearest genes.

HCEs are frequently found in relatively gene poor regions [3], and their distances are conserved among the mammalian genomes [5]. Our data show that IHRs shared by the six vertebrates are also enriched in gene poor regions of their genomes. CpG islands are generally associated with human promoters [18] and most promoter-associated CpG islands that have been reported are located within 2 Kb regions around transcription start sites [19,20]. The enrichment of CpG islands in the IHRs over the random background genomic regions suggests the possibility of the existence of potential target genes, and the long distance between the IHRs and the nearest gene indicates that putative targets might be located in a wider genomic range, or that the CpG islands residing in the IHRs along with the two side HCEs could together perform important roles either as regulatory blocks or other unknown functions.

## Discussion

Out data suggests that subsets of HCE pairs may undergo different evolutionary paths for their genomic distance conservation. We also examined a few features for the functional regions constituted by HCEs and their interior or adjacent sequences, and found that the precise spacing of HCEs to be an important aspect of the HCE structures. Highly conserved structural relationship of HCEs among genomes [5] indicates the feasibility that HCEs are not independent and that two or more HCEs may function together with adjacent sequences as a combined unit. We are not the first to propose the viewpoint that a portion of genomic region function as a united block. Chromosomal segments termed "genomic regulatory blocks (GRBs)" have been annotated in the human genome, formed by conserved relationships between HCEs and their assumed target genes [10]. Higher-order functional architecture also illuminates functional domain structure of the ENCODE regions [9].

Early observations of HCEs strongly suggested their function as acting on vertebrate *cis*-regulatory elements (cREs) of early developmental genes [2,21,22], however cREs are not necessarily strongly conserved but have been regarded as more 'evolvable' than coding sequences [23]. Highly complex correlations between HCEs and their putative target genes also question the idea that the primary function of HCEs is as cis-regulatory elements [24]. Recently, function as "counting units" has been suggested to be associated with such elements [11]. Overlapping, multiple functions have been suggested by several studies to account for the extreme sequence conservation of HCEs [4]. HCE-rich regions are reported to be associated with histone methylation [25]. Increasing evidence suggested their functional association with chromatin remodeling accompanying the involvement of HCEs in other functions like cREs. Most intergenic IHRs are located far away from annotated protein coding genes. Both long distance and relatively close related associations between HCEs and genes were identified [24]. If the IHRs contain elements for chromatin structure and thus perform epigenetic regulation of gene transcription, this would either indicate a form of long distance regulatory action, or that other functional elements (not protein coding genes) are associated with these IHRs, or that the IHRs are *per se *functional units independent of target genes.

We detected 188 IHR1s with extremely conserved distances among deeply divergent species. Distance conservation between highly divergent organisms implies the extreme constraint on the evolution of the IHR1 lengths, which strongly suggests that their distances are functionally important. One possible interpretation for the less conservation of IHR2 lengths would be that some functional elements were inserted or deleted in the IHR2s, or alternatively, the expanded distance is indeed the requirement of difference in their potential biological function among genomes.

## Conclusion

In this study, the bimodal distribution profiles of RDD values still persisted when mapping the integrated data set of HCEs onto the five non-mammalian genomes. We detected two groups of genomic regions confined by HCE pairs with distinct distance conservation pattern in vertebrate genomes. The data suggests these IHRs may function as combined unit, and that subsets of IHRs with distinct space conservation should be treated differently.

## Methods

### Data

Genome sequences were downloaded from UCSC GoldenPath database for the 7 species: human (hg18), mouse (mm7), rat (rn4), chicken (galGal2), frog (xenTro2), zebrafish (danRer3), tetraodon (tetNrg1) and fugu (fr1). UCE [1] and CNE [2] dataset were obtained from the respective authors. The UCR [3] dataset was obtained from . The TFR (>5 kb) data set was obtained from [12]. The collections of annotated genes, the transposon, the repeat and CpG island annotation files for the human genome were downloaded from UCSC GoldenPath database . Collections of pair wise orthologous groups between human and other genomes were downloaded from the InParanoid database [26].

The three datasets of conserved elements were integrated together. Using the human genome as the reference, we extended physical loci to the most remote start/end positions of those elements that have intersections with each other, and we obtained 7,570 highly conserved elements (HCEs) without overlap.

### Assignment of unique homologous HCE hits

The human HCE sequences were mapped onto the rodent (mouse and rat) and five non-mammalian vertebrate genomes (chicken, frog, zebrafish, fugu and tetraodon) with non-stringent BLASTn parameters (mismatch penalty -1, gap open penalty 1, word size 9, and soft masking). Hits for each HCE with an e-value ≤ 10^-5 ^were considered to be under constraints of sequence conservation and kept for further analysis.

A number of HCEs have multiple BLASTn hits in the non-mammalian genomes [see Additional file 19]. To determine which hit is potentially the orthologous one is difficult with only sequence similarity information. The relative order of UCEs along the chromosomes has been found to be nearly identical among mammalian genomes, at a level similar to that of genes and strong conservation of mutual distances among vertebrate species was also found [5]. Thus, criterions of consecutiveness and distance conservation were added to locate the HCEs uniquely onto the non-mammalian genomes [see Additional file 2]. For the cases where some HCEs have multi-alignment hits and some have no BLASTn hit in the query genome, two hits were looked as one pair according to the query genome, if there are less than two other HCEs located in between the two consecutive HCEs in the non-human genomes. RDD [5] values were calculated to measure the conservation of distance between the HCEs pairs. The pairs which were unique in the non-mammal genome were kept, and were divided into three categories according to their linkage relationship with other HCE pairs or associated orthologous genes. For the HCEs with multi-BLASTn hits pairs, we treat them as the corresponding HCEs in the non-mammal genomes on the condition of linkage with other HCE pairs or orthologous genes. Because HCEs tend to be located in clusters, linkage condition of HCE pairs is the first screening step. Thus, the corresponding |RDD| value might not be the minimum. If there were no existing linkage, the two consecutive HCEs with minimum |RDD| value were kept and thus positioned the corresponding HCEs in the query genome.

### Assignment of homologous element pairs

Two HCEs or genes were regarded as a conserved pair if they were found as neighbors in the genomes of both (or all) the species compared [see Additional file 20].

### Calculation of relative distance differences between HCE pairs

To investigate the conservation of distances between the HCEs pairs, we used the same definition as presented in our previous work [5], i.e. RDD = (d_q_-d_h_)/[(d_q_+d_h_)/2]; d_q _and d_h _being the distance between the mid-points of two HCEs of a pair in the query (non-human) and human genomes [see Additional file 20], respectively.

### Partitioning HCE pairs into two groups

By using R clustering function 'pam', we partitioned 403 HCE pairs shared by the five pair wise genomes intro 2 groups based on their |RDD| values dissimilarity matrix. The 'pam' algorithm is a more robust version of K-means, and it is based on the search for 'k' (the number of clusters specified, we let k equal to 2) representative objects or medoids among the observations of the dataset. These observations should represent the structure of the data. After finding a set of 'k' medoids, 'k' clusters are constructed by assigning each observation to the nearest medoid. The goal is to find 'k' representative objects, which minimize the sum of the dissimilarities of the observations to their closest representative object.

We limited inter-HCE regions (IHRs) with boundary marked by the HCE pairs by removing HCEs themselves.

### Random sampling

We randomly selected the same number of regions with same size as IHRs as a negative control. To evaluate the statistical significance of the features of the IHRs, analysis was repeated 1,000 times with independent, randomly sampled data sets. The fraction of times in which the random sample sets had higher (or lower) average scores than those of the IHRs provided the basis for the statistical significance.

Statistical analyses were carried out using the R language [27].

## Authors' contributions

HS designed the study, performed bioinformatics analyses and wrote the manuscript. GS contributed to the manuscript. XZ collected the data. WL edited the manuscript. YL designed and sponsored the study. All authors read and approved the manuscript.

## Supplementary Material

Additional file 1 **Overlap between HCEs from different data sets.** Click here for file

Additional file 2 **The flowchart to assign unique homologous HCE hits in the query genomes.** Click here for file

Additional file 3 **Number of HCE pairs in each category defined under a series of screening constraints.** Click here for file

Additional file 4 **Cumulative percentage of HCEs with increasing number of linked HCEs.** Click here for file

Additional file 5 **RDDs between HCE, gene and exon pairs of the five non-mamalian species compared with human.** Click here for file

Additional file 6 **RDD distributions of three sets of data.** Click here for file

Additional file 7 **|RDD| range within the percentage range of HCE pairs.** Click here for file

Additional file 8 **Number of HCE pairs in each category according to human – non-mammalian pair wise comparison.** Click here for file

Additional file 9 **Genomic distance between HCE pairs.** Click here for file

Additional file 10 **Location of IHRs in the human genome (hg18).** Click here for file

Additional file 11 **Relative distance differences for HCE pairs in pair wise genome comparisons.** Click here for file

Additional file 12 **Frequency of distances between intergenic IHRs and the nearest genes in the human genome.** Click here for file

Additional file 13 **Length of genomic regions containing same group of IHRs and the number of IHRs in the region.** Click here for file

Additional file 14 **Overlap between IHRs and TFRs.** Click here for file

Additional file 15 **Frequency of random regions with human INDEL variations.** Click here for file

Additional file 16 **Number of IHRs containing CpG islands and the percentage of CpG islands' length.** Click here for file

Additional file 17 **The number of IHRs intersected with CpG islands.** Click here for file

Additional file 18 **Number of IHRs containing CpG islands.** Click here for file

Additional file 19 **Copy number of human HCEs in seven genomes.** Click here for file

Additional file 20 **A sketch map of genomic distance between conserved HCE pairs.** Click here for file
