# Supplementary material for: Genomic regions with distinct genomic distance conservation in vertebrate genomes
Source: BMC Genomics. 2009 Mar 27;10:133. doi: 10.1186/1471-2164-10-133 (PMC2667192; doi:10.1186/1471-2164-10-133)
Supplement: Additional file 1 — Overlap between HCEs from different data sets. [file 1471-2164-10-133-S1.pdf]

**Additional file 1:** Overlap between HCEs from different data sets [1].

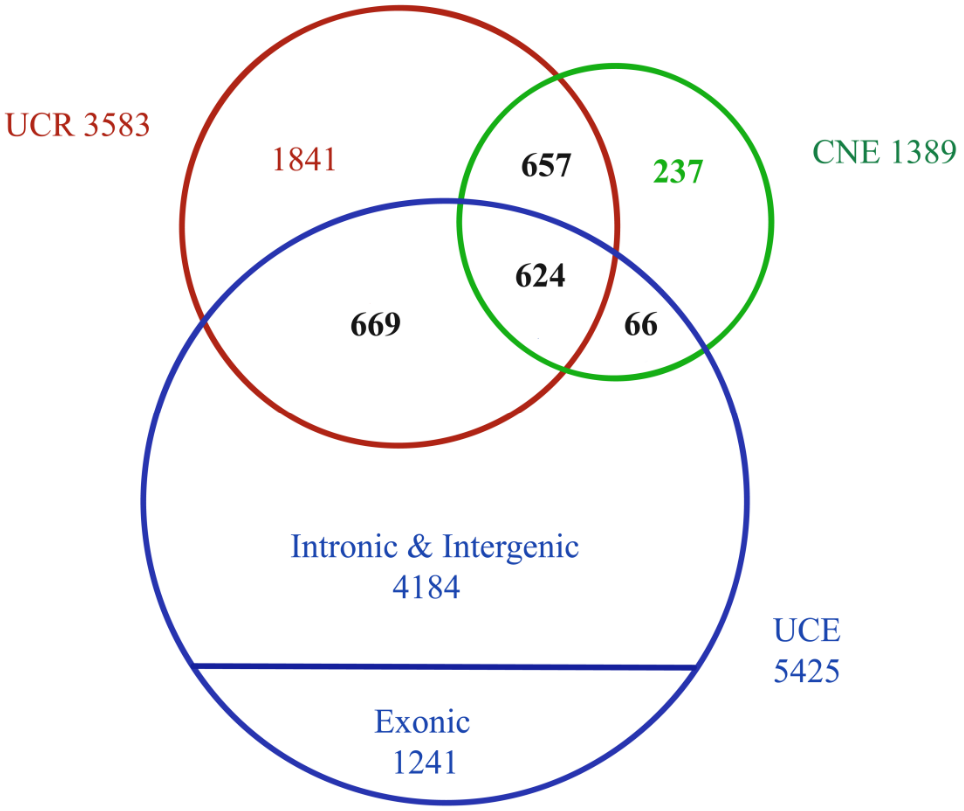


The figure shows UCRs (red, [2]), CNEs (green, [3]) and UCEs (blue, [4]) with at least partial (more than 1 bp) overlap in the human genome.

**References**


1. Sun H, Skogerbo G, Chen R: **Conserved distances between vertebrate highly conserved elements**. *Hum Mol Genet* 2006, **15**(19):2911-2922.
2. Sandelin A, Bailey P, Bruce S, Engstrom PG, Klos JM, Wasserman WW, Ericson J, Lenhard B: **Arrays of ultraconserved non-coding regions span the loci of key developmental genes in vertebrate genomes**. *BMC Genomics* 2004, **5**(1):99.
3. Woolfe A, Goodson M, Goode DK, Snell P, McEwen GK, Vavouri T, Smith SF, North P, Callaway H, Kelly K *et al*: **Highly conserved non-coding sequences are associated with vertebrate development**. *PLoS Biol* 2005, **3**(1):e7.
4. Bejerano G, Pheasant M, Makunin I, Stephen S, Kent WJ, Mattick JS, Haussler D: **Ultraconserved elements in the human genome**. *Science* 2004, **304**(5675):1321-1325.
