# Supplementary figures and images for: Genomic regions with distinct genomic distance conservation in vertebrate genomes
Source: BMC Genomics. 2009 Mar 27;10:133. doi: 10.1186/1471-2164-10-133 (PMC2667192; doi:10.1186/1471-2164-10-133)

**Additional file 2:** The flowchart to assign unique homologous HCE hits in the query genomes.

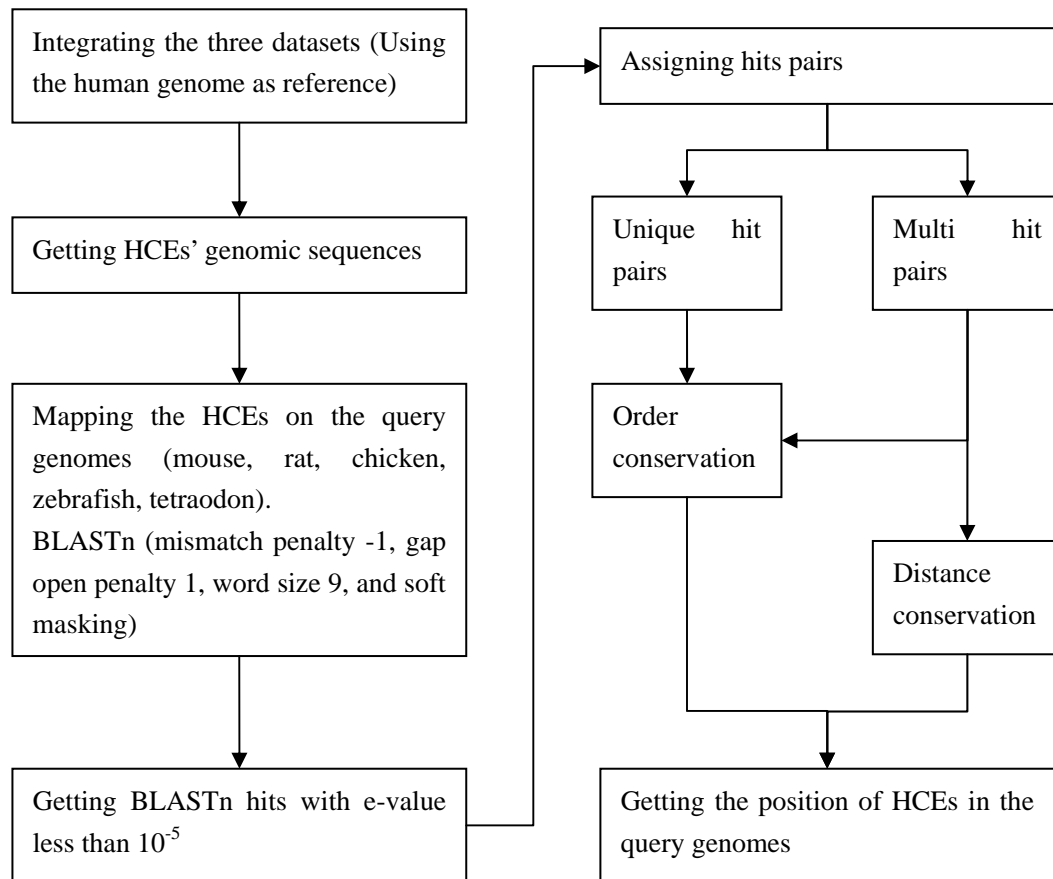

Supplement: Additional file 2 — The flowchart to assign unique homologous HCE hits in the query genomes. [file 1471-2164-10-133-S2.pdf]

**Additional file 6:** RDD distributions of three sets of data (HCE, gene and exon)

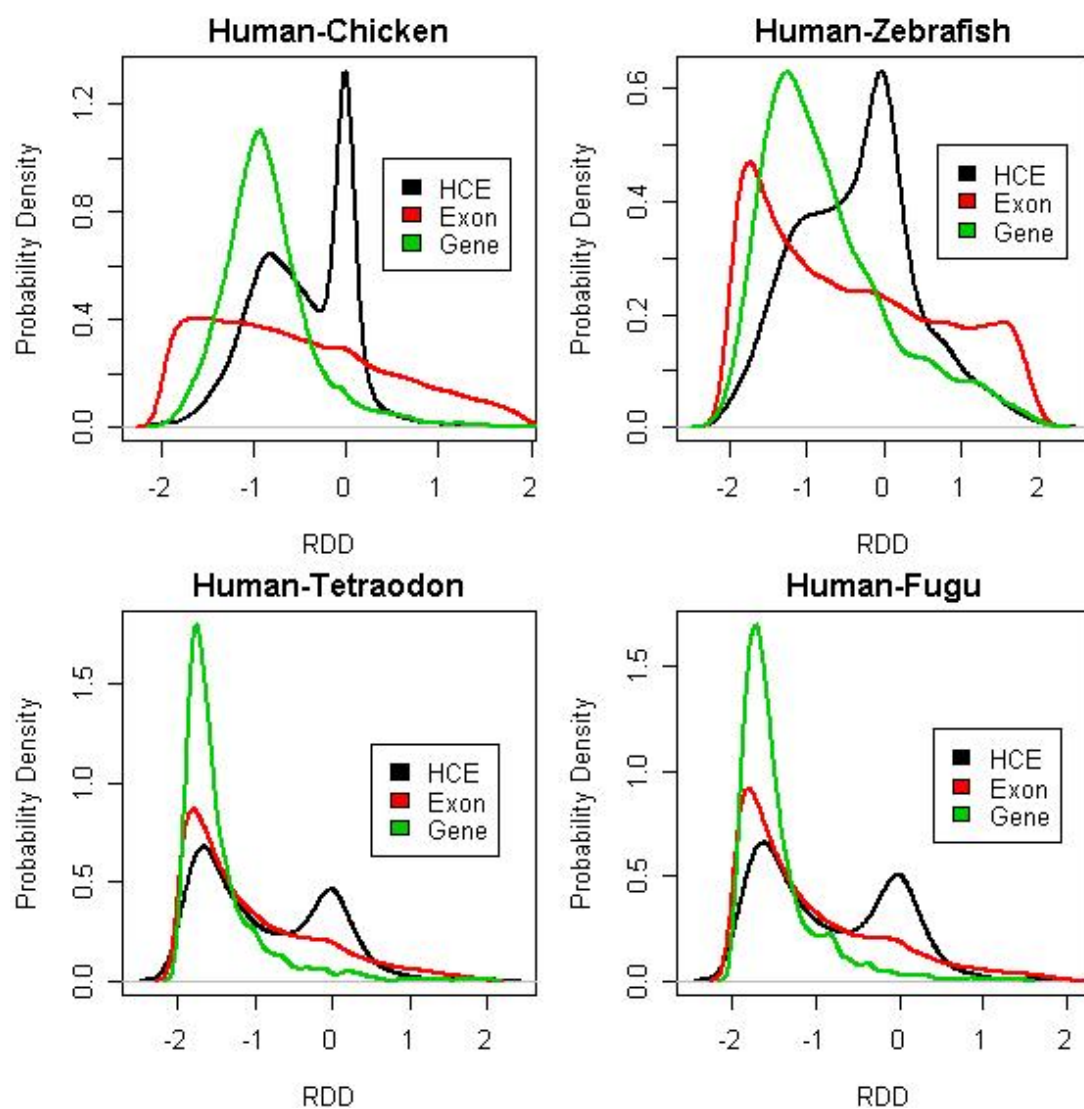

Supplement: Additional file 6 — RDD distributions of three sets of data. [file 1471-2164-10-133-S6.pdf]
