# Supplementary material for: Genomic regions with distinct genomic distance conservation in vertebrate genomes
Source: BMC Genomics. 2009 Mar 27;10:133. doi: 10.1186/1471-2164-10-133 (PMC2667192; doi:10.1186/1471-2164-10-133)
Supplement: Additional file 3 — Number of HCE pairs in each category defined under a series of screening constraints. [file 1471-2164-10-133-S3.pdf]

Additional file 3: Number of HCE pairs in each category defined under a series of screening constraints (Additional file 2).

		Chicken		Frog		Zebrafish		Tetraodon		Fugu	
		Number	(%)	Number	(%)	Number	(%)	Number	(%)	Number	(%)
S	SA	4442	94.39	3155	88.95	836	59.71	1037	81.33	1236	76.53
	SB	29	0.62	0	0	0	0	0	0	0	0
	SC	16	0.34	132	3.72	117	8.36	98	7.69	181	11.21
	Total S	4487	95.35	3287	92.67	953	68.07	1135	89.02	1417	87.74
M	MA	198	4.21	220	6.20	381	27.21	112	8.78	140	8.67
	MB	15	0.32	24	0.68	16	1.14	10	0.78	19	1.18
	MC	5	0.11	0	0	0	0	0	0	0	0
	MD	1	0.02	16	0.45	50	3.57	18	1.41	39	2.41
	Total M	219	4.65	260	7.33	447	31.93	140	10.98	198	12.26
Total HCE pairs		4706	100	3547	100	1400	100	1275	100	1615	100
Total HCEs		5385	71.1	4470	59.0	2015	26.6	1831	24.2	2323	30.7

S: two HCEs of a pair are unique in the query genome; **SA:** there are at least one HCE pair linked in the query genome as they do in the human genome; **SB:** no other HCE pairs are linked but they are linked with orthologous genes; **SC:** neither other HCE pairs nor orthologous genes linked with them. **M:** one or two HCEs of a pair have multi BLASTn hits; **MA:** there are at least one HCE pair linked in the query genome as they do in the human genome, with minimum |RDD|; **MB:** there are at least one HCE pair linked in the query genome as they do in the human genome, with hyper-minimum |RDD|; **MC:** no other HCE pairs are linked but there are orthologous genes linked with them; **MD:** neither other HCE pairs nor orthologous genes linked, with minimum |RDD|.
