# Supplementary material for: Genomic regions with distinct genomic distance conservation in vertebrate genomes
Source: BMC Genomics. 2009 Mar 27;10:133. doi: 10.1186/1471-2164-10-133 (PMC2667192; doi:10.1186/1471-2164-10-133)
Supplement: Additional file 4 — Cumulative percentage of HCEs with increasing number of linked HCEs. [file 1471-2164-10-133-S4.pdf]

Additional file 4: Cumulative percentage of HCEs with increasing number of linked HCEs

	≥ 2	≥ 3	≥ 4	≥ 5	≥ 6	≥ 7	≥ 8	≥ 9	≥ 10
Mouse	99.7	99.3	97.7	97.5	96.8	96.4	95.7	95.2	94.6
Rat	99.6	99.1	97.5	97.1	97.5	93.7	92.9	91.8	90.8
Chicken	99.9	98.4	97.5	96.5	95.3	93.7	92.1	90.0	88.2
Frog	99.8	93.7	89.1	85.0	80.2	77.3	74.8	72.4	70.3
Zebrafish	99.6	82.4	73.7	64.7	58.0	52.1	46.3	40.4	36.7
Tetraodon	99.5	83.9	75.6	69.7	64.9	57.7	54.9	50.3	46.6
Fugu	99.4	81.1	70.5	62.4	56.7	50.7	44.6	40.8	37.1
