# Supplementary material for: Genomic regions with distinct genomic distance conservation in vertebrate genomes
Source: BMC Genomics. 2009 Mar 27;10:133. doi: 10.1186/1471-2164-10-133 (PMC2667192; doi:10.1186/1471-2164-10-133)
Supplement: Additional file 5 — RDDs between HCE, gene and exon pairs of the five non-mamalian species compared with human. [file 1471-2164-10-133-S5.pdf]

Additional file 5: RDDs between HCE, gene and exon pairs of the five non-mamalian species compared with human.

			Human-Chicken	Human-Frog	Human-Zebrafish	Human-Tetraodon	Human-Fugu
**HCE-HCE**	No. of pairs		4706	3547	1400	1275	1615
	RDD	Median	-0.4274	-0.1690	-0.2944	-1.0930	-1.0140
		Mean	-0.4587	-0.2715	-0.3407	-0.8890	-0.8483
	|RDD|	Median	0.4641	0.4635	0.5666	1.1010	1.0320
		Mean	0.5168	0.5545	0.6558	0.9836	0.9438
**Exon-Exon**	No. of pairs		80638		34267	50585	48964
	RDD	Median	-0.6776		-0.6006	-1.3190	-1.3610
		Mean	-0.5294		-0.4118	-1.0380	-1.0750
	|RDD|	Median	0.9472		1.1190	1.3430	1.3840
		Mean	0.9569		1.0660	1.2000	1.2280
**Gene-Gene**	No. of pairs		6375		604	1673	1833
	RDD	Median	-0.9054		-0.9329	-1.6220	-1.6170
		Mean	-0.8480		-0.7298	-1.4670	-0.4730
	|RDD|	Median	0.9125		1.0400	1.624	1.617
		Mean	0.8999		0.9741	1.4930	1.4920
p* value (HCE,Exon)			2.2e-16		2.2e-16	2.2e-16	2.2e-16
p* value (HCE,Gene)			2.2e-16		2.2e-16	2.2e-16	2.2e-16
p* value (Exon,Gene)			2.3e-12		6.4e-06	2.2e-16	2.2e-16

* Wilcoxons unpaired test was used to test the significance.
