# Supplementary material for: Genomic regions with distinct genomic distance conservation in vertebrate genomes
Source: BMC Genomics. 2009 Mar 27;10:133. doi: 10.1186/1471-2164-10-133 (PMC2667192; doi:10.1186/1471-2164-10-133)
Supplement: Additional file 7 — |RDD| range within the percentage range of HCE pairs. [file 1471-2164-10-133-S7.pdf]

Additional file 7: |RDD| range within the percentage range of HCE pairs.

Percentage ≤	Human-Chicken	Human-Frog	Human-Zebrafish	Human-Tetraodon	Human-Fugu
68 %	0.766	0.766	0.896	1.505	1.467
50 %	0.465	0.464	0.568	1.102	1.032
40 %	0.275	0.303	0.387	0.758	0.651
30 %	0.116	0.169	0.251	0.409	0.349
