# Supplementary material for: Genomic regions with distinct genomic distance conservation in vertebrate genomes
Source: BMC Genomics. 2009 Mar 27;10:133. doi: 10.1186/1471-2164-10-133 (PMC2667192; doi:10.1186/1471-2164-10-133)
Supplement: Additional file 8 — Number of HCE pairs in each category according to human – non-mammalian pair wise comparison. [file 1471-2164-10-133-S8.pdf]

Additional file 8: Number of HCE pairs in each category according to human – non-mammalian pair wise comparison (see definition of categories in Additional file 3).

Categories		Query genomes				
		Chicken	Frog	Zebrafish	Tetraodon	Fugu
S	SA	352	324	226	319	303
	SB	1	0	0	0	0
	SC	5	16	24	22	30
	Total	358	240	250	341	333
M	MA	34	51	131	50	43
	MB	7	7	5	5	8
	MC	4	0	0	0	0
	MD	0	5	17	7	19
	Total	45	63	153	62	70
