# Supplementary material for: Genomic regions with distinct genomic distance conservation in vertebrate genomes
Source: BMC Genomics. 2009 Mar 27;10:133. doi: 10.1186/1471-2164-10-133 (PMC2667192; doi:10.1186/1471-2164-10-133)
Supplement: Additional file 9 — Genomic distance between HCE pairs. [file 1471-2164-10-133-S9.pdf]

Additional file 9: Genomic distance between HCE pairs (bp).

	Min	Median	Mean	Max	Total
Group one	2	575	2783	114900	523255
Group two	650	25640	46090	693200	9909207
Total	2	6764	25890	693200	10432462

The human genome was used as baseline. Wilcoxon unpaired test was used to test the significance of the difference between the two groups of IHRs' genomic distances ($p < 2.2e^{-16}$).
