# Supplementary material for: Genomic regions with distinct genomic distance conservation in vertebrate genomes
Source: BMC Genomics. 2009 Mar 27;10:133. doi: 10.1186/1471-2164-10-133 (PMC2667192; doi:10.1186/1471-2164-10-133)
Supplement: Additional file 11 — Relative distance differences for HCE pairs in pair wise genome comparisons. [file 1471-2164-10-133-S11.pdf]

Additional file 11: Relative distance differences (|RDD|) for HCE pairs in pair wise genome comparisons.

	Human		Mouse		Rat		Chicken		Zebrafish		Tetraodon		Fugu	
	Median	Mean	Median	Mean	Median	Mean	Median	Mean	Median	Mean	Median	Mean	Median	Mean
Human	-	-	0.1463	0.2098	0.1731	0.3552	0.6221	0.6349	0.9025	0.9149	1.558	1.4792	1.5837	1.4927
Mouse	0.0149	0.0638	-	-	0.0709	0.2824	0.5277	0.592	0.8828	0.8968	1.5254	1.448	1.5518	1.4626
Rat	0.0171	0.0748	0.0072	0.0775	-	-	0.5807	0.6983	0.9751	0.9864	1.5375	1.4854	1.5657	1.5035
Chicken	0.0438	0.1459	0.0537	0.1779	0.0475	0.1921	-	-	0.6354	0.7333	1.2225	1.162	1.233	1.2041
Zebrafish	0.2533	0.4681	0.2737	0.4829	0.2586	0.4929	0.3091	0.5131	-	-	1.0941	1.0621	1.0585	1.073
Tetraodon	0.2397	0.3612	0.2717	0.3895	0.2596	0.3919	0.2588	0.3796	0.365	0.5665	-	-	0.0535	0.1973
Fugu	0.2152	0.3951	0.2428	0.4212	0.24	0.425	0.2416	0.4073	0.3364	0.6009	0.0423	0.1827	-	-

Down triangle matrix for HCE pairs of IHR1 and up triangle matrix for HCE pairs of IHR2.
