# Supplementary material for: Genomic regions with distinct genomic distance conservation in vertebrate genomes
Source: BMC Genomics. 2009 Mar 27;10:133. doi: 10.1186/1471-2164-10-133 (PMC2667192; doi:10.1186/1471-2164-10-133)

Additional file 12: Frequency of distances between intergenic IHRs and the nearest genes in the human genome. (Black bar: all IHRs; blue bar: IHRs with CpG islands).

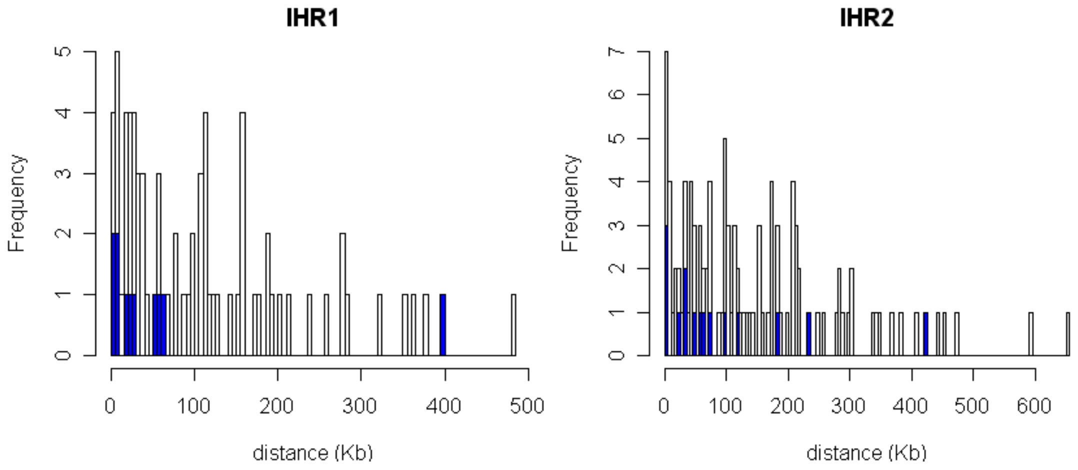

Supplement: Additional file 12 — Frequency of distances between intergenic IHRs and the nearest genes in the human genome. [file 1471-2164-10-133-S12.pdf]
