# Supplementary material for: Genomic regions with distinct genomic distance conservation in vertebrate genomes
Source: BMC Genomics. 2009 Mar 27;10:133. doi: 10.1186/1471-2164-10-133 (PMC2667192; doi:10.1186/1471-2164-10-133)
Supplement: Additional file 13 — Length of genomic regions containing same group of IHRs and the number of IHRs in the region. [file 1471-2164-10-133-S13.pdf]

Additional file 13: Length of genomic regions containing same group of IHRs and the number of IHRs in the region.

Number of IHRs		1	2	3	4	5	6	7	8	9	10
IHR1	Number of cases	58	26	6	4	2	0	1	1	1	1
	Length (Kb)	1.8	5716	23093	11655	129	-	105099	29456	41	902
IHR2	Number of cases	49	22	20	4	4	2	2	0	0	0
	Length (Kb)	37.4	5260	7272	4932	2901	17161	80354	-	-	-
