# Supplementary material for: Genomic regions with distinct genomic distance conservation in vertebrate genomes
Source: BMC Genomics. 2009 Mar 27;10:133. doi: 10.1186/1471-2164-10-133 (PMC2667192; doi:10.1186/1471-2164-10-133)
Supplement: Additional file 14 — Overlap between IHRs and TFRs. [file 1471-2164-10-133-S14.pdf]

Additional file 14: Overlap between IHRs and TFRs.

	IHR1			IHR2		
	Observed	p* value (<)	Expected	Observed	p* value (<)	Expected
Number of IHRs intersected with TFR(s)	112 (60%)	0.001	5 (2.6%)	112 (52%)	0.001	27 (12%)
Average percentage of TFR length over the IHR	90%	0.15	73%	27%	0.014	18%

* Randomly selected human genomic regions were used to test the significance, the fraction of times in which the random sample set average scored greater than the average of the IHRs provided the basis for the statistical significance.
