# Supplementary material for: Genomic regions with distinct genomic distance conservation in vertebrate genomes
Source: BMC Genomics. 2009 Mar 27;10:133. doi: 10.1186/1471-2164-10-133 (PMC2667192; doi:10.1186/1471-2164-10-133)

Additional file 15: Frequency of random regions with human INDEL variations (insertion and deletion).

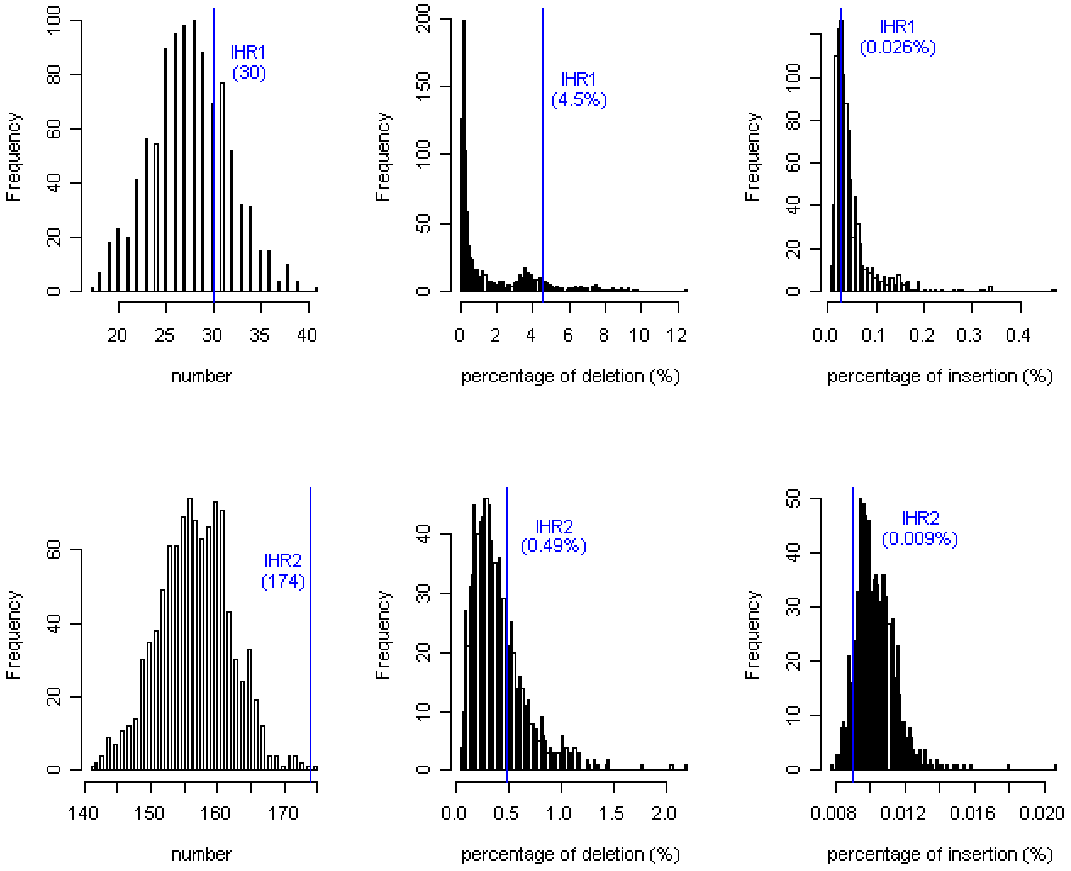

Supplement: Additional file 15 — Frequency of random regions with human INDEL variations. [file 1471-2164-10-133-S15.pdf]
