# Supplementary material for: Genomic regions with distinct genomic distance conservation in vertebrate genomes
Source: BMC Genomics. 2009 Mar 27;10:133. doi: 10.1186/1471-2164-10-133 (PMC2667192; doi:10.1186/1471-2164-10-133)
Supplement: Additional file 16 — Number of IHRs containing CpG islands and the percentage of CpG islands' length. [file 1471-2164-10-133-S16.pdf]

Additional file 16: Number of IHRs containing CpG islands and the percentage of CpG islands' length.

	IHR1		IHR2	
	Observed	Expected	Observed	Expected
Percentage of IHRs contain CpG island (%)	10.6 (p* = 0.001)	0.5	14.4 (p* = 0.001)	2.3
Average percentage of CpG islands' length (%)	44.7 (p* = 0.11)	13.6	7.1 (p* = 0.06)	3.5

* Randomly selected human genomic regions were used to test the significance; p value was given in the bracket. Two sample Wilcoxon test was used to test the significance of difference in the percentage of CpG islands length between IHR1s and IHR2s: p = 5.5e-6
