# Supplementary material for: Genomic regions with distinct genomic distance conservation in vertebrate genomes
Source: BMC Genomics. 2009 Mar 27;10:133. doi: 10.1186/1471-2164-10-133 (PMC2667192; doi:10.1186/1471-2164-10-133)

Additional file 17: The number of IHRs intersected with CpG islands is significantly greater than regions selected randomly corresponding to the same size and length (p value < 0.001).

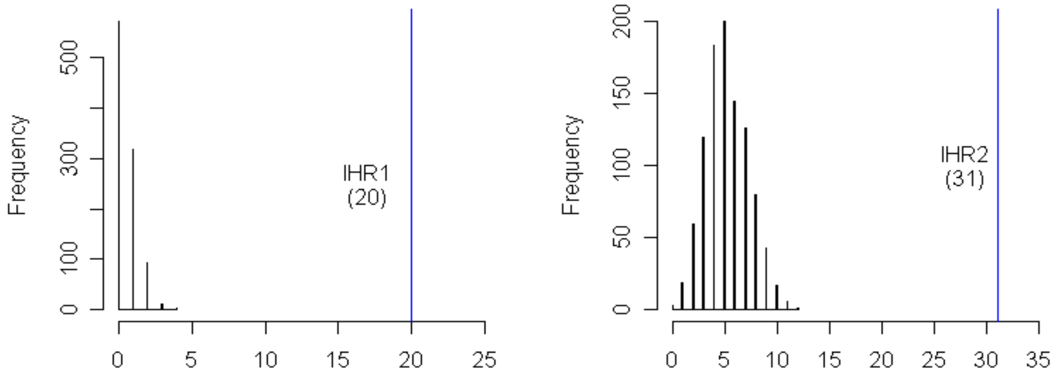

Supplement: Additional file 17 — The number of IHRs intersected with CpG islands. [file 1471-2164-10-133-S17.pdf]
