# Supplementary material for: Genomic regions with distinct genomic distance conservation in vertebrate genomes
Source: BMC Genomics. 2009 Mar 27;10:133. doi: 10.1186/1471-2164-10-133 (PMC2667192; doi:10.1186/1471-2164-10-133)
Supplement: Additional file 18 — Number of IHRs containing CpG islands. [file 1471-2164-10-133-S18.pdf]

Additional file 18: Number of IHRs containing CpG islands.

Pair wise genomic location	IHR1			IHR2		
	total	with CpG islands	p* value	total	with CpG islands	p* value
Exonic-Exonic	29	4	0.8260	16	4	0.9388
Intronic-Intronic	62	1	0.0024	57	0	2.9E-05
Intergenic-Intergenic	77	11	0.9432	111	17	0.7187
Exonic-Intronic	16	2	0.7667	12	1	0.4608
Exonic-Intergenic	4	2	0.9961	4	2	0.99
Intronic-Intergenic	0	0	1	15	7	0.9997
total	188	20		215	31	

* Hypergeometric test was used to test the enrichment.
