# Supplementary material for: Genomic regions with distinct genomic distance conservation in vertebrate genomes
Source: BMC Genomics. 2009 Mar 27;10:133. doi: 10.1186/1471-2164-10-133 (PMC2667192; doi:10.1186/1471-2164-10-133)
Supplement: Additional file 19 — Copy number of human HCEs in seven genomes. [file 1471-2164-10-133-S19.pdf]

Additional file 19: Copy number of human HCEs in seven genomes.

		mouse		rat		chicken		frog		zebrafish		fugu		tetraodon	
		O	E	O	E	O	E	O	E	O	E	O	E	O	E
HCE	Single	7150	6419	6962	6261	5137	5025	4474	4588	1867	2701	2668	3061	2173	2372
	Multi	314	1044	319	1019	706	817	861	746	1274	439	892	498	586	386
	Total	7464		7281		5843		5335		3141		3560		2759	
	Multi/Total (%)	4.2	13.9	4.4	13.9	12.1	13.9	16.1	13.9	40.5	13.9	25.0	13.9	21.2	13.9

Chi-squared contingency table test was used to calculate the expected copy number and test the significance. **O** stand for observed copy number and **E** for expected. (X-squared = 3515.043, df = 6, p-value < 2.2e-16)
