# Supplementary material for: Genomic regions with distinct genomic distance conservation in vertebrate genomes
Source: BMC Genomics. 2009 Mar 27;10:133. doi: 10.1186/1471-2164-10-133 (PMC2667192; doi:10.1186/1471-2164-10-133)
Supplement: Additional file 20 — A sketch map of genomic distance between conserved HCE pairs. [file 1471-2164-10-133-S20.pdf]

Additional file 20: A sketch map of genomic distance between conserved HCE pairs.

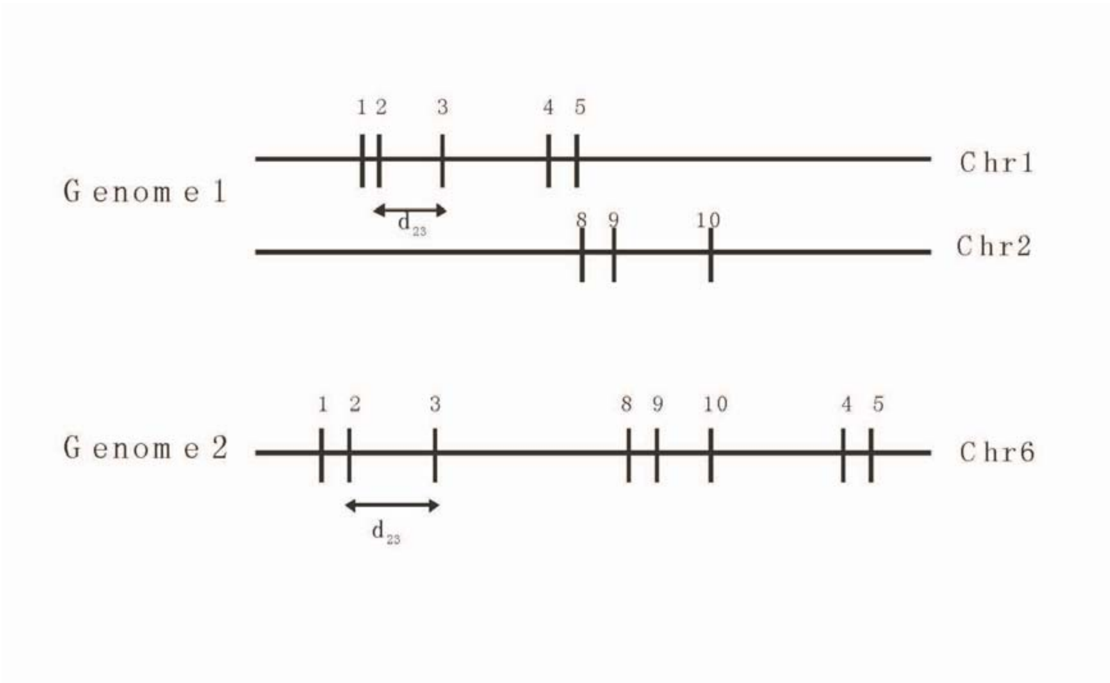

In this example, five HCE pairs are conserved between the two genomes (e.g. HCE1-HCE2, HCE2-HCE3, HCE4-HCE5, HCE8-HCE9 and HCE9-HCE10). HCE3 and HCE4 are not a pair since they are not consecutive neighbors in genome 2. Similarly, HCE3 and HCE8, HCE10 and HCE4 are not pairs as they are located on two different chromosomes in genome 1. The genomic distance of HCE2-HCE3 pair was illustrated in the cartoon.
